# How Far Do the Facts Accompanying the Prevalence of Epidemic Cholera in Chicago, during the Summer and Autumn of 1866, Throw Light on the Etiology of That Disease

**Published:** 1867-11

**Authors:** N. S. Davis

**Affiliations:** Professor of Principles and Practice of Medicine, and of Clinical Medicine, Chicago, Ill.


CHICAGO MEDICAL EXAMINER.

N. S. DAVIS, M.D., Editor.

VOL. VIII. NOVEMBER, 1867. NO. 11.

### Original Contributions.

ARTICLE XLVII.

### HOW FAR DO THE FACTS ACCOMPANYING THE PREVALENCE OF EPIDEMIC CHOLERA IN CHICAGO, DURING THE SUMMER AND AUTUMN OF 1866, THROW LIGHT ON THE ETIOLOGY OF THAT DISEASE? *Presented to the Section on Meteorology, Medical Topography, and Epidemic Diseases of the American Medical Association, May, 1867.*

By N. S. DAVIS, M.D., Professor of Principles and Practice of Medicine, and of Clinical Medicine, Chicago, Ill.

At the meeting of the American Medical Association, in May, 1866, I read a brief paper before the Section on Meteorology, Topography, and Epidemics, in which attention was called to the alleged fact, that all the diseases usually regarded as *epidemic* could be arranged into two classes. The first class embraces all such as arise directly from a specific virus or animal poison, capable of being reproduced in the living animal body; and, consequently capable of being propagated from individual to individual in any climate, at any season of the year, and without regard to public or individual hygienic conditions. The chief members of this class are, Variola, Varicella, Rubeola, and Scarlatina. The second class was represented as embracing such diseases as appear in an epidemic form only at irregular intervals, prevail only for a limited period of time, and at any given prevalence appear incapable of propagation beyond certain geographical limits or sanitary conditions. Influenza, diphtheria, spotted fever, erysipelas, cholera, etc., were enumerated as examples of this class. In specifying the essential differences between these two classes of epidemics, in that paper, it was stated that all the members of the second class had their analogues or typical forms among the ordinary sporadic and endemic diseases of every season. It was said that epidemic "influenza has its type in the severer cases of coryza and catarrh; cholera, in the cholera-morbus of every returning summer; diphtheria, in the folicular and erythematic inflammations of the fauces; and erysipelas, in the sporadic and traumatic cases of common occurrence." Assuming such a relation to exist between these sporadic and epidemic forms of disease, it was asked whether it would not be more in accordance with the acknowledged rules of inductive reasoning, as well as more likely to lead to important and reliable results, to look for the essential causes of this class of epidemics among those circumstances and influences that are already recognized as influencing their sporadic types?

For the purpose of illustrating further, both the connection between certain sporadic and epidemic diseases, and the local causes influencing their development, I have prepared the following summary of facts observed during the year 1866. The prevalence of cholera in Europe, during the summer of 1865, led to a general apprehension that the same disease would prevail more or less in our country during the succeeding year. Influenced by a desire to gain possession of every fact that would throw light on the etiology of that disease, more especially as regarded local influences, I commenced early in the year 1866 to make an almost daily record of the atmospheric and local conditions in the City of Chicago, in connection with the prevalence and special character of diseases, and continued such record throughout the year. To enable others to appreciate the bearing of certain facts, more especially in regard to the direction and amount of winds or atmospheric currents, it should be stated that the city occupies a very level prairie on the western border of Lake Michigan. The soil is an alluvial mixture of clay, sand, and the products of vegetable decay, and is closely underlaid by a stratum of tenacious blue clay, impervious to the surface water. A wide expanse of similar low, wet prairie skirts the City on the west and south, while the open waters of Lake Michigan extend forty miles eastward, and several hundred miles north and north-east. The streets of the City are broad, straight, and cross each other at right angles, thereby affording great facilities for ventilation by atmospheric currents. Only a very few families live in basements or apartments below the surface of the ground. Winds from the south and west, passing over a wide expanse of alluvial prairie, are very cold in winter; but warm, relaxing, and more or less impregnated with malaria, or the products of vegetable decomposition, during the summer. Winds from the north and east, passing over a wide expanse of pure water, are less intensely cold in winter, but always cool, bracing, and often even chilly in summer. The general level condition of the surface, both of the City and the surrounding country, with the impervious stratum of clay subsoil, causes much surface water to be retained until returned to the atmosphere by evaporation. Consequently, dampness is a marked feature of our climate. During the year 1865, the inhabitants of Chicago enjoyed a greater immunity from fatal sickness than usual, the ratio of mortality being slightly below the average for the preceding five years. The only prominent meteorological peculiarity of that year was an unusually cold and wet summer, with high winds. The gross mortality of the City, during 1865, was 3633, the population being 187,000, gave the ratio of one death to 50, or 20 in every 1000. Of the 3633 deaths, in 1865, 612 were attributed to diseases of the bowels, namely: from inflammation of the bowels 76, dysentery 85, cholera-morbus 51, diarrhœa in adults 83, diarrhœa in children under five years of age 317. From the first of January to the first of June, 1866, the same general exemption from unusual sickness and mortality continued, as during the preceding year. The gross mortality was: for January 293, February 260, March 254, April 278, and May

275. During the months of April and May, the atmosphere was comparatively cool, dry, with abundant free electricity, and a prevalence of north, north-east, and north-west winds. The only noteable exception to these general conditions, was on the 19th of May, when a direct south wind brought a current of hot, damp atmosphere, extremely oppressive, but which was followed by showers in the evening, and a cool north wind in the morning. The wind continued from the north and north-west, with a cool, dry atmosphere, and an average electrical and ozonic indication until the night of the 7th of June. The morning of the 8th was ushered in with wind from the south, and a hot, sultry, and oppresive atmosphere all day. The atmosphere was damp, and deficient in both electricity and ozone. Slight showers fell during the evening, and before the morning of the 9th, the wind changed to the west. The 9th and 10th were cool, dry, and pleasant, with winds from the north and north-east. On the morning of the 11th, the wind again changed to the south, and before mid-day, the atmosphere was more hot and oppressive than on the 8th. It continued so throughout the 11th and 12th, with showers on the evening of both days. On the 13th, the wind varied from the south to the west, with less heat, but returned to the south on the morning of the 14th, with increased heat, and copious showers of rain from the south-west in the afternoon.

During these four days the atmosphere was damp, hot, and below the ordinary indications of free electricity and ozone. The sensible effect upon the healthy animal economy, was that of relaxation, lassitude, and indisposition to exertion. In popular language it is expressed in the words, "sultry and oppressive." From the morning of the fifteenth to the twentieth, the wind was continuously from the north and the north-east. The atmosphere was cool, clear, dry, and gave indications of free electricity and ozone equal to the average. On the twentieth, the wind again changed to the south, and remained south and south-west until the twenty-seventh. During these days the mornings were almost uniformly cool and clear, but the afternoons hot and oppressive. They were dry, except the afternoon of the twenty-first, when there were showers of rain with lightning. During the last three days of the month, the atmosphere was cool, for the most part clear and dry, with winds from the north and north-east.

Comparing these dates with my notes on the daily attacks of disease, it appears that the first notable increase of bowel-affections took place during the eleventh and twelfth, when the atmosphere was hot, damp, and oppressive. During these two days, a large number of cases of diarrhœa, cholera-morbus, and dysentery dated their origin. Far the larger number of cases occurred in children under three years of age. It was during the night of the eleventh that the first two cases, presenting all the ordinary symptoms of epidemic cholera, were observed. One was a woman residing at 212 Kankakee Avenue, in the extreme south-eastern part of the city, while the other was a man on West Harrison Street, near the western limits of the City. (See map.) From the fourteenth to the twentieth, but very few new cases of either diarrhœa or cholera-morbus occurred. But from the twenty-first to the twenty-seventh, these diseases again increased so rapidly, that my notes on the twenty-sixth and twenty-seventh show one-half of the whole number of patients prescribed for to have had either diarrhœa, cholera-morbus, or dysentery. The total mortality in June was 319, being an increase of 45 over the preceding month, and of 126 over the corresponding month of 1865.

During the first seven days of July, the winds were from the south and south-west; the temperature high; more or less rain every day, except the fourth and fifth; atmosphere excessively damp and deficient in electricity, inducing in the animal system a feeling of languor and oppression. The only change during these seven days, was on the evening of the second, when the wind suddenly changed to the north-west, bringing a cooler atmosphere during the night, but which was again reversed in the morning. On the eighth, the wind had changed to the north-east, and the atmosphere continued chilly and damp through the day. Ninth, wind from the north, and the atmosphere cool and clear, but less damp. Tenth and eleventh, wind south-east, atmosphere warm, but clear, dry, and pleasant. From the morning of the twelfth to the evening of the seventeenth, the wind was from the south and south-west; the atmosphere continuously hot, damp, and deficient in free electricity. About 1 o'clock, P.M., of the seventeenth, the wind suddenly changed to the north-east, bringing a cool, damp atmosphere, and a slight sprinkle of rain, and in the evening, copious showers, with thunder and lightning. During the eighteenth, nineteenth, and twentieth, the wind continued north and north-east, with a cool, clear, but moderately damp atmosphere. During the evening of the twentieth, it became cloudy, with the wind more to the south. Twenty-first, wind south, cloudy, hot, and oppressive, with copious showers in the evening, followed by a cool north-east wind, and a fine display of atmospheric electricity. At midnight, the wind again changed to the south, and the morning of the twenty-second was hot, rainy, and oppressive. The twenty-third, twenty-fourth, and twenty-fifth, were only moderately warm, and the wind mostly east and north-east, with showers every day or night, keeping the air saturated with dampness. On the twenty-sixth, twenty-seventh, and twenty-eighth, the winds were continuously from the south; the atmosphere filled with clouds, mist, and sometimes rain, and very hot. During the night of the twenty-eighth, the wind changed to the north, and the twenty-ninth, thirtieth, and thirty-first, were cool, clear, and pleasant.

It will thus be seen that the most prominent meteorological characteristics of July were, high temperature; frequent rains; excessive dampness; south and south-west winds, with deficient free atmospheric electricity. The principal exceptions to this rule were from the seventeenth to the twenty-first, and from the twenty-ninth to the thirty-first. The average temperature of the month was here, as in most other sections of our country, above the average of the preceding ten years.

In regard to local sanitary conditions, it is proper to state that, although the scavenger system adopted by the City authorities had served to keep the streets and alleys of the central part of the City some cleaner than in former years, yet up to the last day of July, there were extensive sections of the City, thickly covered with dwellings, in which not even a street-gutter had been cleaned out, and both branches of the Chicago river were in their usually offensive condition. As might have been expected, from the local and atmospheric conditions just detailed, the prevalence of intestinal diseases in the City, during the month of July, was much above the average of the preceding three or four years.

It was stated that from the twentieth to the twenty-seventh of June, there was a prevalence of south winds, with a hot, damp, and oppressive atmosphere, during which, "serous diarrhœa and dysentery increased rapidly among young children, and to some extent among adults." The cool invigorating atmosphere of the last three days of June checked this tendency in some degree, but the renewal of the hot, damp, and showery weather of the first seven days of July, again increased rapidly the number of attacks of cholera-morbus, cholera-infantum, diarrhœa, and dysentery.

During the sixth and seventh, particularly, the attacks of bowel-affections were, not only numerous, but they were accompanied by unusual languor, prostration, and sweating. The days and nights were hot, cloudy, some rain, and slight wind from the south. Two cases of sunstroke were reported on the seventh.

On the night of the sixth and morning of the seventh, I saw four children presenting symptoms of great prostration; countenances pale; pulse slow and weak; respiration slow and inefficient; mental faculties dull, or in a state of semi-coma, from which it was difficult to arouse them; occasional sighing, and momentary restlessness; but with little or no evacuations of any kind. Their paleness and apparent lifelessness was the chief cause of alarm to the parents and friends. I have noticed a few similar cases during the first extremely hot days and nights of almost every summer. They appear to be the result of a deficiency of atmospheric oxygen and electricity, coupled with the relaxing influence of a high temperature. From the eighth to the eleventh, the number of new attacks of bowel-affections was less, and the larger number of those that did occur, presented the form of dysentery. But from the eleventh to the seventeenth, the cases of serous diarrhœa and cholera-morbus, especially among children, increased so rapidly that two-thirds of all the patients who came under my observation during that period, were of this class.

Among my daily entries in the note-book, is the following: "During the night of the sixteenth, the air was very hot and oppressive. Both that and the preceding night, children with bowel-complaints became very restless and prostrate, with discharges thin as water. Several were taken suddenly prostrate, drowsy, and feverish, without any discharges." Other periods of rapid increase of new attacks took place during the twenty-first and twenty-second, and from the twenty-sixth to the twenty-eighth, inclusive. According to my notes, four-fifths of the cases of serous diarrhœa and cholera-morbus, that occurred during the whole month, had their *commencement*, either between the first and seventh, the eleventh and seventeenth, twenty-first and twenty-second, or twenty-sixth and twenty-eighth, while the cases of dysentery occurred more uniform throughout the month. If these dates are compared with the detail of atmospheric conditions, it will be seen that a hot and damp atmosphere invariably accompanied the unusually rapid multiplication of cases of diarrhœa and cholera-morbus. Are these atmospheric conditions, coupled with the idio-miasmatic influences generally present in densely populated cities, capable of causing attacks of genuine spasmodic cholera? As bearing on this question, I will simply relate the following facts:

On the night of the seventh of July, one of the most oppressive that occurred during the month, a lady living at 160 Western Avenue, on the extreme western limits of the City, began to have disturbance of the bowels, with serous discharges, which culminated in severe cholera symptoms on the night of the eighth, including the characteristic rice-water discharges by vomiting and purging; severe cramps in the muscles of the extremities and abdomen; a small weak pulse; skin shrivelled and cool; voice husky, etc. Under appropriate treatment she recovered.

Mr. L., living at 200 Huron Street, in the North Division of the City, commenced having serous diarrhœa on the evening of the seventh, which developed into full severe cholera during the night of the ninth.

On the morning of the eleventh, a lady from Kansas arrived at the Adams House, one of the principal hotels of the City. She had been much fatigued by the long journey, and had suffered from a moderate diarrhœa during the three preceding days. Soon after her arrival, the discharges began rapidly to increase, and at noon, she was presenting every symptom of severe cholera. The discharges, up and down, were copious, rice-water in appearance, and accompanied by severe muscular cramps in the extremities. Two hours later, when I saw her, the eyes were deeply sunken; the extremities cold and skin shrunken; the pulse extremely weak; the voice reduced to a whisper; the cramps severe, and the intestinal discharges wholly involuntary. Under treatment the discharges ceased, reaction took place slowly, and the patient ultimately recovered.

On the afternoon of the twelfth, a baker aged about twenty years, living at 172 West Lake Street, was suddenly attacked with severe symptoms of cholera. I saw him about two hours after the commencement of the attack, and the cholera symptoms were strongly marked in every particular. The day was excessively hot, and the man had indulged in drinking eight or ten glasses of beer, two of brandy, besides considerable water.

During the night of the sixth, or morning of the seventh, Mr. R., aged fifty-five years, living at the corner of West Indiana and Sangamon Streets, was attacked with all the symptoms of violent cholera. He had long been in infirm health, and when I saw him, about noon of the seventh, he was in complete collapse. Partial reaction took place, and he lingered until the tenth, when he died.

Mr. W., aged forty-five, living at 245 Madison Street, was attacked with all the symptoms of cholera, on the night of the fifteenth. I saw him early in the morning of the sixteenth, when the characteristic features of the disease were as perfect as in cases seen in the midst of the epidemic of 1854.

Mr. D., aged thirty-five years, a laboring man of intemperate habits, living on Franklin Street, near Jackson, was attacked during the night of the fifteenth. When I saw him, early in the morning of the sixteenth, he appeared to be in the commencement of collapse. Reaction, however, took place, and secondary fever followed, but he finally recovered.

Early in the morning of the twenty-second, I was called in quick succession to three cases of adults, who had been suddenly attacked during the latter part of the night previous with vomiting, purging, cramps, etc. They were seen during the early part of the active stage, and readily yielded to treatment.

On the twenty-first, a case occurred at 282 West Chicago Avenue, which was reported to the Board of Health as genuine epidemic cholera, by Dr. Addison.

Such were the indications of cholera among adults in this City during the month of July, as they came under my own observation. During the whole month cholera-infantum was unusually prevalent and fatal. That the month of July, 1866, was attended by a much greater proportion of bowel-affections than usual, is apparent from the very great increase of mortality. The whole number of deaths in July, 1866, was 706; while for the corresponding month of 1865, it was only 425.

The last three days of July were cool, clear, and pleasant.

It remained cool, with a prevalence of north and north-east winds until the morning of the eighth of August. Showers of rain fell on the third, and steady rain all the night of the sixth. On the morning of the eighth, the wind changed to the south; the air was filled with mist or aqueous vapor, and at midday, was very hot and oppressive. Between five and six o'clock in the afternoon, the wind suddenly changed to the north, and the atmosphere became so cool as to produce chilliness. It remained cool and clear until the eleventh, when the wind changed to the south, the sky became cloudy, and in the evening there fell copious showers with sharp lightning. The twelfth was mostly clear, wind south, and atmosphere very warm and damp. These conditions continued until 11 o'clock, A.M., of the thirteenth, when the wind again changed to the north, and the air became cool and clear. But at midnight, the direction of the wind was reversed, and the morning of the fourteenth was hot, damp, and oppressive, followed by showers of rain in the afternoon. From this time to the twentieth, the atmosphere was still, or moved only slightly by winds from the south or south-east, very damp, and moderately warm. During the seventeenth and eighteenth, especially, the air was still, and so light that smoke and mist hung close to the earth, instead of rising to the higher regions or drifting away with atmospheric currents.

Rain fell moderately on the evening of the eighteenth and the afternoon of the twentieth. From the twenty-first to the twenty-fifth, the wind was from the north, and the atmosphere cool, even to chilliness at times. Rain fell nearly all of the twenty-third. From the morning of the twenty-sixth to the thirty-first, the atmosphere was again still, damp, and moderately warm, very similar to that from the fourteenth to the twentieth. It was filled with smoke and mist, especially during the nights. The slight winds that were felt, came from the south or south-west, except during the afternoon of the twenty-eighth, when it blew from the north-east, and was cooler.

Slight rains fell on the twenty-ninth and thirtieth, and in the evening of the thirty-first, copious showers fell, with *thunder* and *lightning*, during which the wind changed to the north-west, and blew a stiff breeze. Vivid flashes of lightning continued until a late hour of the night. It was the first display of the kind during the month. From the first to the twenty-fifth of September, the weather was almost continuously cool and rainy, with a prevalence of north and north-east winds. The rains were so frequent and copious that the surface of the earth was kept constantly saturated with fresh fallen water. Occasionally, the sun would shine out clear and warm for two or three hours in the middle of the day; but during the whole time named, there were not three consecutive warm, dry days.

From the twenty-sixth of September, however, to the seventh of October, the atmosphere was clear, moderately warm, and pleasant.

The prevalent winds were from the north, north-west, and north-east. On the seventh, the wind changed to the south and became very light. From that time to the thirteenth, the atmosphere was filled with mist and smoke, and so still as to be scarcely moved by a breeze either day or night. The sky was, most of the time, clear and the atmosphere warm. On the thirteenth, a light breeze sprang up from the north-east, the air was more heavy, and the mist and smoke that had so steadily filled the lower strata of the atmosphere, were dissipated in twenty-four hours. From this to the nineteenth, the weather was mostly clear and pleasant, though warm, and very little wind from any quarter. On the twentieth, there came a strong south west wind, accompanied by clouds and some rain. During the afternoon and evening, the wind became a severe gale, and blew down the walls of some unfinished houses in the City, and during the night, copious showers fell, accompanied by vivid lightning. The twenty-second was cold, mostly clear, with a strong west wind, and frost at night. The twenty-third was cold, cloudy, with a light fall of snow, sufficient to whiten the side-walks.

During the first seven days of August, attacks of cholera-infantum and serous diarrhœa were less frequent than in the middle of July. On the eighth, there was a sudden increase of these affections, and a still more marked increase on the twelfth, when I met one case of cholera at 109 North Water Street, and another on Wabash Avenue, south of Twelfth Street. On the sixth of August, a company of Mormons left one of their number, a native of Denmark, sick at the Rail Road Depot, from whence he was taken to the County Hospital, located between Eighteenth and Nineteenth Streets, where he died in the evening of the same day, with all the symptoms of epidemic cholera. On the ninth, three days after the Dane died, a female nurse, and a male inmate of the Hospital were attacked. From that time, one or more cases occurred daily among the inmates until the eighteenth, when it ceased. The whole number of cases occurring in the institution was 19, of whom 11 recovered and 8 died.

Nearly simultaneous with this outbreak, in the County Hospital, cases of cholera occurred in almost every section of the City. Two were known to occur on the twelfth: one was at 109 North Water Street, and the other on Wabash Avenue, south of Twelfth Street. One case was reported on the thirteenth, at 61 East White Street. Five on the fourteenth, namely: at 241 Monroe Street, 239 Illinois Street, 63 Michigan Avenue, and 323 South Morgan Street; and fifteen on the fifteenth, namely: at 261 and 288 South Wells, 4 Wendell, 43 Quincy, 33 Pierce, 109 and 115 North Water, 125 Superior, 218 DeKoven, 461 South Clark, Alley rear 169 Polk, 65 Indiana, Tremont House, man from St. Louis, and 62 South Canal Street.

From this date to the twentieth, new cases occurred daily in all parts of the city. From the twentieth to the twenty-sixth, the number of cases diminished, but again moderately increased during the remainder of the month. From the first to the twentieth of September, the disease continued in pretty uniform rate of prevalence, although it was restricted more closely to the population of foreign birth, and in parts of the City least improved by drainage and cleanliness. During the two weeks intervening between the twenty-third of September, and the fifth of October, the number of attacks diminished to such a degree that the disease was regarded by many practitioners in the city, as substantially extinct.

The whole number of deaths attributed to cholera during the month of August was 139, and September 166, or an average of five deaths per day. On the seventh of October, however, it was apparent that the disease was again rapidly increasing, seven deaths having been reported on that day. Cases continued to multiply rapidly until the twelfth when the number of deaths, daily, were about 30. From this date until the severe storm of wind and rain on the twenty-second, the disease gradually declined, and then suddenly ceased. The whole number of deaths attributed to cholera during the month of October was 673, being an average of nearly twenty-two per day.

The City is divided into sixteen wards, and though the cholera prevailed in all of them, such prevalence was very unequal.

Thus, of 1550 cases reported to the Board of Health, during the months of August, September, and October, 474, or nearly one-third of the whole, were in the Seventh, Fourteenth, and Sixteenth Wards, while only 130 were in the Fifth, Eighth, and Ninth. The localities of its prevalence will be better appreciated by reference to the accompanying map. The deep red lines bound the several wards. The Seventh may be said to constitute the centre of the Irish laboring population, while the Fourteenth bears the same relation to the Germans.

The number of cases of cholera reported to the Board of Health from the Seventh, was 1 per every 100 inhabitants, and in the Fourteenth, it was 1 for every 95 of population. While in those sections, inhabited almost exclusively by Americans, the ratio reported did not exceed 1 in 250 of the population.

The special districts of cholera prevalence, cannot be accurately represented by the wards, and hence, they are better defined on the map by deep blue lines.*

The predilection of the disease for certain localities was not more apparent, than for the different classes of people and nationalities. The total population of Chicago is nearly equally divided between natives of the United States and those of foreign birth. But of 1500 cases of cholera, concerning which the nativity was given, 287 were natives of the United States;

* The reader will better understand the topography of Chicago if we explain that the highest, most sandy and dry parts of the City lie between Clark Street and the Lake shore, both in the North and South Divisions, and in the Ninth and Tenth Wards, in the West Division. These portions are occupied almost wholly by the best class of residences, and most thoroughly drained by permanent sewers.

The lowest and most alluvial districts skirt both sides of the North and South Branches of the River; but more especially between the east bank of the North Branch and North Wells Street, in the North Division, and between the west bank of the South Branch of the River and South Halsted Street, south of the line of Van Buren Street, in the West Division. These districts are not only low and alluvial, naturally, but they are only partially intersected by sewers, and are covered pretty thickly by a laboring population of foreign birth.

The great business centre of the City is embraced in the First Ward, but extends into the Sixteenth Ward, north, and into the Tenth and Eleventh Wards, west.

546 of Germany; 373 of Ireland; 98 of Norway; 45 of Sweden; 42 of England; 34 of Bohemia; 15 of Canada; 13 of Scotland; 9 of Holland; 9 of France; 9 of Denmark; 7 of Finland; 6 of Belgium; 4 of Switzerland; 3 of Italy; 4 of Poland; 1 of Prussia; and 1 of Portugal.

The total mortality for 1866 was 5937, of which 990 were attributed to epidemic cholera, and 1176 to other bowel-affections. Ratio of deaths to population, 1 to 35.

A careful examination of all the facts thus far set forth, will fully establish the following propositions:

1st. That the cause, or causes which gave rise to the unusual prevalence of bowel-affections, in Chicago, during the year 1866, commenced their influence in June, and continued it until the end of October.

This is strikingly exhibited by the following table:

| | 1865. Total Mortality. | 1865. Cholera. | 1865. Other Bowel-Affections. | 1866. Total Mortality. | 1866. Cholera. | 1866. Other Bowel-Affections. |
|---|---|---|---|---|---|---|
| June, | 197 | 0 | 20 | 319 | 0 | 45 |
| July, | 425 | 0 | 163 | 706 | 2 | 297 |
| [illegible]ugust, | 464 | 0 | 208 | 940 | 139 | 376 |
| [illegible]eptember, | 346 | 0 | 112 | 739 | 166 | 205 |
| [illegible]ctober, | 360 | 0 | 66 | 1170 | 673 | 114 |
| [illegible]ovember, | 299 | 0 | 40 | 382 | 12 | 28 |

2nd. That cholera, cholera-morbus, and diarrhœa, in both [illegible]ildren and adults, were uniformly increased by high atmos[illegible]heric temperature and moisture, coincident with south or south-west winds, and the surface of the earth *moist*, that is, neither covered with fresh fallen water nor completely dry.

3rd. That the epidemic cholera, especially, manifested a striking predilection for, and adherence to, such localities as pres[illegible]nted the greatest dampness, coupled with the greatest accu[illegible]ulation of decomposable animal and vegetable matter, with [illegible]e least facilities for ventilation and drainage.

4th. That neither in its beginning, progress, or decline, could [illegible]ere be traced any influence from the importation of persons [illegible] goods from other localities.

For instance, it began in persons who had resided in Chicago for several years; in localities remote from railroad depots, docks, hotels, and warehouses, and in persons who had not been at work in such places; (see cases at 212 Kankakee Avenue, and corner of West Harrison and Morgan Streets, in June, 160 Western Avenue, corner of West Indiana and North Sangamon Streets, 282 West Chicago Avenue, and others in July;) and the disease was altogether most severe and fatal in those sections of the City remote from connection with the centres of trade and travel (see Seventh and Fourteenth Wards, on the map.)

The first case that could be supposed to have brought the disease from some other locality, was the Dane who was taken from the railroad depot to the County Hospital, on the sixth of August. But if we look to this individual for the introduction of the disease, how shall we account for the occasional cases presenting all the phenomena of cholera, and the extraordinary mortality from bowel-affections during the whole of July preceding?

Again, if this individual introduced the infection, why did it not extend first among the large laboring population immediately around the Hospital, in the Third Ward, instead of appearing almost simultaneously in a dozen remote portions of the City, and in persons who had had no possible communication with either the Hospital or its inmates?

By what law of diffusion did it leap over the intervening thousands from the Hospital on Eighteenth Street, in the Third Ward, to light on three children in an alley out of White Street, in the Fifteenth Ward, and with such violence that they were all dead within twenty-four hours? Or, how, during the same day, did it get by everything in front, and search out a poor, secluded old woman, living in a dirty, damp, old house in the rear of 288 South Wells Street? We might go on multiplying such questions indefinitely. It has been said by those who regard the essential cause of cholera as existing in the dejections, that the disease is often spread unsuspectingly by "walking" cases of the disease; that is, by persons having true cholera diarrhœa, but not so severe as to deter them from going about or travelling, and consequently depositing the cholera poison, wherever they chance to deposit their intestinal evacuations. Unfortunately for this theory, however, many of the early cases of cholera, occurring in this City, in the recent epidemic were in localities so remote or secluded that no stranger or traveller in the city would have had the remotest chance of entering them either by accident or otherwise. On the other hand, it frequently happened throughout the whole season, that a case of violent cholera would occur in a boarding-house, or a hotel, and remain until death or recovery, without imparting the disease to a single other occupant of the same premises during the season. For instance, early in August, a man coming direct from St. Louis, was attacked with cholera on the cars, and on his arrival he was taken in a hack to the Tremont House, where he died the same day. Yet no other case followed it in the same hotel. Indeed, according to my records, a large majority of the cases that came under my personal observations, presented but a single case in the same house. The circumstances, however, which seem to illustrate most strikingly the influence of local causes on the prevalence of this disease, occurred in the latter part of the season.

As already stated, the month of September was characterized by almost constant rains from the first to the twenty-fourth of the month. The consequence was that the level and only partially drained portions of the City, became flooded with fresh fallen water. This was particularly the case in the Sixth, Seventh, Eleventh, Twelfth, and the parts of the Fourteenth, Fifteenth, and Sixteenth Wards, lying between Wells Street and the North Branch of the River. These sections of the city are covered with small wooden houses, occupied chiefly by a population of foreign birth. All of these have out-of-door privies, and many of them board shanties for stabling a horse or cow, or both, on the rear end of the lots. Very few of the privy-vaults are water-tight. Consequently the copious rains of September caused hundreds of these to overflow with water, carrying the fluid part of the contents, with the mascerations of the manure heap into the surface soil of the whole lot. While the flood of fresh fallen water continued, no deleterious consequences were developed. On the contrary, the prevalence of cholera steadily declined from the fifteenth to the twenty-fifth of the month. So much so, that at the latter date, its prevalence was very generally regarded as ended. Its prevalence in a severe epidemic form had also ceased in the neighboring cities of Cincinnati and St. Louis. On the twenty-fourth of September, the rains ceased, and the atmosphere became clear, cool, and pleasant, and remained so until the end of the month. In the meantime, the Annual State Agricultural Fair opened in the City on the twenty-fourth, and during the six succeeding days was freely visited by thousands, coming from every part of the State. Yet all this company of strangers came and went wholly unharmed, so far as as sickness was concerned. In the meantime, the excess of fresh surface water had been dissipated, and to a casual observer, streets and lots looked dry and pleasant.

During the first week in October, the sky continued clear, the atmosphere was warmer, and scarcely moved by a breeze for several days; emenations began to rapidly impregnate the air from the surface soil so recently saturated with water holding in solution the contents of privies, etc., and by the end of the week, cholera was again doing its work of death with threefold greater rapidity than at any previous time during the summer. Can there be any doubt about the relation between causes and effects here? There had been no trace of any fresh importation of the disease from other cities or towns; and yet, under certain conditions of soil and atmosphere, it attains the proportions of a perfect epidemic almost in a day. Thus developed, its ravages continue until between the twenty-first and twenty-third of the month, when these same conditions become suddenly and violently reversed, attended by an equally sudden disappearance of the disease.

It may be said that the cholera infection, or "seeds," had been previously scattered throughout the City, and that the local and atmospheric conditions described, only served to multiply and impart activity to the previously existing poison. If we admit this, it still leaves in full force the fact of paramount practical value, that whatever may be the origin of the supposed cholera infection, or *germs*, the safety of every community depends upon its own atmospheric and sanitary conditions. Without entering into the domains of theoretical controversy, I will only add the opinion that whenever the origin, progress, and termination of cholera epidemics shall be carefully and minutely studied in their relation to the preceding and accompanying local and atmospheric conditions, and to the prevalence of sporadic cases, with other bowel-affections, it will be found that the same causes that are now acknowledged to be necessary to give *activity* to the supposed infection, are also capable of *originating* it.

